# Doctrines on Exposed Pulps

**Published:** 1862-10

**Authors:** W. H. Atkinson


### DOCTRINES ON EXPOSED PULPS.

BY DR. W. H. ATKINSON.

One would think that geographical limits would not be lines of demarkation of surgical doctrines or practices. If certain localities were more favorable to the development of science than others, a clue to cause of difference might be had. But that differences of doctrines and treatment do exist to a degree confined to localities is very evident from the writings and teachings of our dental practitioners of at least three localities, that may almost be certainly designated by the tenor of their speaking and writing upon the vexed question of exposed dental pulps, viz.: Philadelphia, New York, and the West.

Philadelphia practice and teachings are like young surgeons anxious to be thorough, and hence run into reckless destruction of parts that might, under the light of experience and earnest patience, be preserved.

New York presents the aspect of bold, ambitious and somewhat scientific *cunning* practice, but like most commercial communities, is not quite always reliable. This locality has much skill, some science, but lacks the requisite probity and moral stamina to come together and lead, as they doubtless should, and but for this lack, would to a higher and more truly professional stasis.

Distrust of each other and a sense of imperfection, combined with a desire to pass for more than their par value, has thus far deprived that metropolis of the association and interchange of knowledges on a purely fraternal, democratic basis necessary to establish firmly their claim to (their normal position of) superiority.

The West is young, bold and badly mixed, but not satisfied short of the highest attainment in its reach, and is well inoculated with the ambition to excel, if not actually lead, the older and more pretending localities. Here success and failure alike are permitted to see the light. This is well, for many times a single failure will open our eyes far more than the finest success many times repeated and recorded with the greatest *care.*

I have not designated the first focalizing point of dental teaching as a locality in which certain doctrines and destructive practices took their origin, for the very reason that that locality has been more the point of collecting and correlating than originating doctrines and practices.

This last and greatest dental instructor must soon revive, or no longer rank first, for the elements are astir. For my own part, I could sincerely wish that there were not only the Baltimore, Cincinnati and Philadelphia dental colleges, but that at least each State in the Union possessed efficient means of producing a corps of such operators and teachers as the times imperatively demand.

As yet, it would be difficult to form a code of doctrines as to what is and what is not correct scientific practice in a given routine of cases. And yet the very same physiology and pathology, the same histology and anatomy, the same chemistry and laws of life do underlie and should dictate what is and also just what is not compatible with true professional procedure. Here, as elsewhere, when differences of doctrines and practices prevail, we may be certain we have not arrived at demonstration on both sides.

---

[We are rather at a loss to know what Dr. A. means when he speaks of New York dentists being nominally in a position of superiority; a pretty strong claim, Doctor. Perhaps we can't see it just right. If he intends to say that the dentists of that locality are superior, we demur—we admit they are just as good; but even that is not because they have lived in New York, but in some cases, at least, because they came from some other place. Or if he means that that locality is *superior* in its advantages for the development of dentists, we object, decidedly.

It is true that in a large city there are advantages not found in less thickly settled places, but then there are also great disadvantages, which in many cases preponderate. The truth is, that in all departments of human occupation, the strong men—the men of might—are not produced in large cities—the supply is kept up from some other source.—Ed.]

